# Stochastic errors vs. modeling errors in distance based phylogenetic reconstructions

**DOI:** 10.1186/1748-7188-7-22

**Published:** 2012-08-31

**Authors:** Daniel Doerr, Ilan Gronau, Shlomo Moran, Irad Yavneh

**Affiliations:** 1 Center for Biotechnology, Bielefeld University, Bielefeld, Germany; 2 Department of Biological Statistics and Computational Biology, Cornell University, Ithaca, USA; 3 Computer Science Department, Technion - Israel Institute of Technology, Haifa, Israel

**Keywords:** Phylogenetic reconstructions, Substitution models, Additive substitution rate functions

## Abstract

**Background:**

Distance-based phylogenetic reconstruction methods use evolutionary distances between species in order to reconstruct the phylogenetic tree spanning them. There are many different methods for estimating distances from sequence data. These methods assume different substitution models and have different statistical properties. Since the true substitution model is typically unknown, it is important to consider the effect of model misspecification on the performance of a distance estimation method.

**Results:**

This paper continues the line of research which attempts to adjust to each given set of input sequences a distance function which maximizes the expected topological accuracy of the reconstructed tree. We focus here on the effect of systematic error caused by assuming an inadequate model, but consider also the stochastic error caused by using short sequences. We introduce a theoretical framework for analyzing both sources of error based on the notion of *deviation from additivity*, which quantifies the contribution of model misspecification to the estimation error. We demonstrate this framework by studying the behavior of the Jukes-Cantor distance function when applied to data generated according to Kimura’s two-parameter model with a transition-transversion bias. We provide both a theoretical derivation for this case, and a detailed simulation study on quartet trees.

**Conclusions:**

We demonstrate both analytically and experimentally that by deliberately assuming an oversimplified evolutionary model, it is possible to increase the topological accuracy of reconstruction. Our theoretical framework provides new insights into the mechanisms that enables statistically inconsistent reconstruction methods to outperform consistent methods.

## Introduction

Phylogenetic reconstruction is the task of determining the topology of an evolutionary tree underlying a given set of samples (species) using sequence data extracted from them. This is typically done by assuming some simplified model for DNA sequence evolution, in most cases modeling it as a homogeneous continuous-time Markov process [1-3]. Distance-based reconstruction algorithms tackle this task by first computing a set of n2 pairwise distances between the *n* input samples and then finding a tree which fits these distances. The distance measures used for this purpose typically reflect the rates of certain substitution events along the evolutionary paths in question. We thus refer to these distance measures as *substitution rate (SR) functions*. The distance-based approach is based on the fact that if the SR function used is *additive* for the underlying substitution model, and the input sequences are sufficiently long, then the topology of the true tree can be efficiently recovered with high probability. However, since the underlying evolutionary model is usually unknown, this assumption is rarely satisfied in practice.

Substitution models used for phylogenetic reconstruction range from the simplest Jukes-Cantor (JC) model [4], through slightly more complex and flexible models, such as Kimura’s two-parameter (K2P) model [5] and the Hasegawa-Kishino-Yano model (HKY) [6], to the General Time-Reversible (GTR) model [7,8]. In previous works [9,10] we observed that substitution models which are not too restrictive or too general have many inherently different additive SR functions. We used this basic observation to demonstrate that it is possible to adjust for each given set of DNA sequences a “good” additive SR function, which leads to significantly increased phylogenetic reconstruction accuracy, compared to other additive SR functions. This exploits our ability to predict the *stochastic noise* associated with each SR function. When the SR function used for distance estimation is additive for the underlying substitution model, this stochastic noise is the only cause for inaccurate reconstruction. However, in the scenario, which is very common in practice, where the SR function in use is not additive for the model, an additional systematic bias is introduced in the distance estimates. This systematic bias in distance estimation results in a phylogenetic reconstruction method that might be statistically inconsistent in some cases. In this paper^a^, we extend our previous line of research to this scenario, by removing the constraint of additivity. We do this by considering both the stochastic noise and systematic error.

Several previous studies have demonstrated the utility of phylogenetic reconstruction methods that are not generally statistically consistent. The maximum parsimony method has been long known to be inconsistent in some cases [11,12]. However, in other cases it was shown to be more likely to produce accurate reconstructions, compared with the maximum likelihood method [13-15]. More recently, it has been demonstrated that reconstruction accuracy can be improved by deliberately assuming an oversimplified substitution model, when reconstructing a tree using maximum likelihood [16,17]. In the context of distance-based reconstruction, non-additive distance measures have been shown in several cases to lead to improved accuracy when compared with additive measures [18,19]. Overall, these studies provide convincing evidence for the need to consider inconsistent phylogenetic reconstruction methods. However, none of them provide a rigorous framework for characterizing the cases in which inconsistent methods outperform consistent ones.

In this paper we develop a theoretical framework which provides a practical and systematic way to quantify the effect of distance-estimation-bias on the accuracy of distance-based reconstruction. This framework is based on a novel method for measuring the *deviation from additivity* of SR functions. Coupled with the results in [9], this method enables evaluation of both the systematic bias and stochastic noise of SR functions. Such evaluation is important, because there is often a tradeoff between these two sources of error, stemming from the fact that simpler models with fewer parameters (such as JC) have smaller stochastic noise at the expense of greater estimation bias. Our framework allows us to consider this tradeoff when deciding which SR function to use for a given data set. This allows us to characterize a wide range of cases in which an SR function associated with an oversimplified evolutionary model results in increased reconstruction accuracy.

This finding falls in line with previous studies demonstrating the usefulness of phylogenetic reconstruction methods that are not generally consistent. Previous studies have attributed the increased accuracy of inconsistent methods mainly to the fact that these methods have a bias toward reconstructing certain topologies, leading to increased accuracy in cases where the phylogeny being reconstructed has the “favored topology”. We notice a similar behavior using our theoretical characterization of non-additive SR functions. However, somewhat surprisingly, we find that non-additive SR functions often have an advantage even when the phylogeny being reconstructed has an “unfavorable topology”. This is due to the reduced stochastic noise of the non-additive SR function (compared with it additive alternatives), which compensates for its topological bias.

Our paper is organized as follows. Section “Background” outlines some of the required background and introduces several new concepts that are central in our analysis. Section “Deviation from additivity in homogeneous substitution models” provides the main analytic results in the paper, and introduces *deviation from additivity* as a measure of distance estimation bias. In that section we prove a general upper bound for this deviation and establish a connection with reconstruction accuracy. We then study deviation from additivity and stochastic error of the JC distance formula when applied to data generated under the K2P model. In Section “Performance of Non affine-additive SR functions in quartet resolution” we study the effect of deviation from additivity and stochastic error on the accuracy of quartet reconstruction. In the case of quartets we can draw a tight connection between the different sources of error in distance estimation and inaccuracy of reconstruction. We present a useful heuristic, based on the so-called Fisher criterion ( [20,21]), for comparing the expected accuracy of two SR functions in this context. In Section “Simulations on Hasegawa’s Tree” we extend our study to larger trees using experiments on simulated data based on the tree obtained by Hasegawa in [6]. Finally, In Section “Inferring trees from genomic sequences” we demonstrate our approach through a series of experiments reconstructing trees from bacterial gene sequences.

## Background

In this section we provide a brief exposition of DNA substitution models and substitution rate functions used for distance estimation. We concentrate on details essential to this study and refer the reader to a previous paper [9] and standard textbooks [1,2] for a more complete survey.

### Substitution Models

In this work, a DNA substitution model ℳ is simply a set of stochastic 4×4 *transition matrices* closed under matrix product (i.e., **PQ**∈ℳ→**PQ**∈ℳ). These matrices serve to describe the substitution process along evolutionary paths in a phylogenetic tree. All substitution models addressed in this paper are time-reversible [7]. A *model tree* in a time reversible substitution model ℳ, or an ℳ*tree*, is an undirected tree *t* =(*VE* ) in which each edge *e* ∈*E* is associated with a transition matrix ${\text{P}}_{e}\in ℳ$. An ℳ-tree *t* implies an inter-leaf transition matrix ${\text{P}}_{\mathrm{ij}}\in ℳ$ for each pair of leaves {i,j}⊂L(T), namely ${\text{P}}_{\mathrm{ij}}=\underset{e\in \mathrm{pat}h_{T}(i,j)}{\prod }{\text{P}}_{e}$. Most common models are defined using *rate matrices*, which are 4×4 matrices whose off-diagonal elements are non-negative *substitution rates*, and whose rows sum to 0. A stochastic transition matrix **P** is obtained from a rate matrix **R** through matrix exponentiation: **P**=^*e***R**^.

A common assumption made on the substitution process is that it is *homogeneous* throughout time. This means that all rate matrices in the model are proportional to each other. Such a substitution model is thus termed *homogeneous*, and it is defined by a *unit rate matrix***R** as follows: ${ℳ}_{R}=\{e^{t\text{R}}:t>0\}$. Note that the definition of the unit rate matrix associated with a given homogeneous model is somewhat arbitrary ^b^, but once the unit **R** is defined, it implies a bijection (or equivalence) between rate matrices in ${ℳ}_{R}$ and the parameter *t*, which corresponds to evolutionary time. We will make use of this equivalence extensively throughout this paper.

We use the Kimura’s two-parameter (K2P) model [5] as a concrete example for demonstrating our approach. A rate matrix in this model is defined by two rate parameters: *α*, which is the rate of *transition* -type (ti) substitutions ( A⇔G, C⇔T), and *β*, which is the rate of *transversion* -type (tv) substitutions ( {A,G}⇔{C,T}). Each K2P rate matrix can be represented as a product of a unit rate matrix, in which *α* + 2*β* =1, and a scalar *t* corresponding to *evolutionary time* .

(1) $$\begin{array}{c} {ℳ}_{\text{K2P}}\;=\;\left\{e^{t{\text{R}}_{\alpha ,\beta }}\;|\;t>0,\alpha \geq \beta >0,\alpha +2\beta =1\right\}\;;\;\\ \;{\text{R}}_{\alpha ,\beta }=\left(\begin{array}{cccc} - & \;\alpha & \beta & \;\beta \\ \alpha & \;- & \beta & \;\beta \\ \beta & \;\beta & - & \;\alpha \\ \beta & \;\beta & \alpha & \;- \end{array}\right) \end{array}$$

Each unit rate matrix of the K2P model defines a homogeneous sub-model, which is identified by its unique transition-transversion (ti-tv) ratio $R=\frac{\alpha }{2\beta }\geq \frac{1}{2}$. The Jukes-Cantor (JC) model [4] is a special homogeneous sub-model of K2P, in which $R=\frac{1}{2}$ (i.e., *α* =*β* ). Although the K2P model is defined in (1) as a union of its homogeneous sub-models, it is important to note that this union is closed under matrix product, implying that K2P adheres to our definition of a proper substitution model. Conversely, some commonly used substitution models, such as GTR and HKY, are defined as a union of homogeneous models, but are not themselves closed under matrix product [22].

Transition matrices in the K2P model have the same symmetric structure as the underlying rate matrices, with two distinct transition parameters: *p*_*α*_ – the probability of a transition-type substitution; *p*_*β*_– the probability of a transversion-type substitution. The transformations between (*α*,*β*,*t* ) and (*p*_*α*_,*p*_*β*_) are given by the following equations:

(2) $$\begin{array}{c} \mathrm{\alpha t}\;=\;-\frac{1}{2}\text{ln}(1-2p_{\beta }-2p_{\alpha })+\frac{1}{4}\text{ln}\;(1-4p_{\beta })\\ \mathrm{\beta t}\;=\;-\frac{1}{4}\text{ln}\;(1-4p_{\beta })\;. \end{array}$$

(3) $$\begin{array}{c} p_{\alpha }\;=\;\frac{1}{4}\left(1+e^{-4\mathrm{\beta t}}-2e^{-2\mathrm{\alpha t}-2\mathrm{\beta t}}\right)\\ p_{\beta }\;=\;\frac{1}{4}\left(1-e^{-4\mathrm{\beta t}}\right)\;. \end{array}$$

### Substitution rate functions

A *substitution rate (SR) function* for a model ℳ is a non-negative continuous function $\Delta :ℳ\to R^{+}$ that maps each transition matrix onto a numerical value of “substitution rate”. An SR function *Δ* induces the following *dissimilarity mapping* over the leaves of an ℳ-tree *t* : $D_{\Delta }^{T}(i,j)=\Delta ({\text{P}}_{\mathrm{ij}})$, for all {i,j}⊂L(T). Of particular interest in phylogenetic reconstruction are *additive* SR functions.

#### Definition 2.1 (Additive SR function)

An SR function *Δ* is said to be *additive* for a substitution model ℳ if for all P,Q∈ℳ, *Δ* (**PQ**)=*Δ* (**P**) + *Δ* (**Q**).

It is often explicitly required that an SR function be additive for the assumed model (see [9]). The evolutionary time, *t*, typically serves as the standard additive measure in most common substitution models. Throughout this study we follow the special case of K2P, focusing on the two SR functions defined below.

(4) $$\begin{array}{cc} \;\Delta_{\text{K2P}}(p_{\alpha },p_{\beta }) & =-\frac{1}{2}\text{ln}\;(1-2p_{\beta }-2p_{\alpha })-\frac{1}{4}\text{ln}\;(1-4p_{\beta })\\ & =\;\mathrm{\alpha t}+2\mathrm{\beta t}\;=\;t\;.\;\;\;\;\;\;\;\;\; \end{array}$$

(5) $$\begin{array}{cc} \Delta_{\mathrm{JC}}(p_{\alpha },p_{\beta }) & =-\frac{3}{4}\text{ln}\;\left(1-\frac{4}{3}(p_{\alpha }+2p_{\beta })\right)\\ & =\;-\frac{3}{4}\text{ln}\;\left(\frac{1}{3}(e^{-4\mathrm{\beta t}}+2e^{-2\mathrm{\alpha t}-2\mathrm{\beta t}})\right). \end{array}$$

The first SR function, *Δ*_K2P_, is the common SR function suggested for the K2P model in [5], and it is clearly additive, as it maps the transition probabilities onto evolutionary time *t* . The second SR function, *Δ*_JC_, maps the transition probabilities onto evolutionary time only in the special case of the JC model where *α* =*β* . Under other homogeneous sub-models of K2P, it is non-additive. This non-additivity is analyzed in details in section Deviation from additivity in homogeneous substitution models.

### Additive metrics, Affine-additive mappings, and Near-additivity

The core idea behind distance-based phylogenetic reconstruction is that a phylogenetic tree *t* can be accurately and efficiently reconstructed from pairwise distances which are *additive with respect to t*[23,24].

#### Definition 2.2 (Additive metric)

A metric *d* defined over the leaf-set *L* (*t* ) of a tree *t* is *t -additive* (or *additive w.r.t t* ), if there exists a positive edge-weighting function $w:E(T)\to R^{+}$, such that for each *i*,*j* ∈*L* (*t* ), $D(i,j)=\underset{e\in \mathrm{pat}h_{T}(i,j)}{\sum }w(e)$. *d* is *additive* for a set *S* if it is *t* -additive for some tree *t* where *L* (*t* )=*S* .

It is well known that additive SR functions imply additive metrics: if *Δ* is an additive SR function for a model ℳ, then for any ℳ-tree *t*, $D_{\Delta }^{T}$ (the dissimilarity mapping induced by *Δ* on *t* ) is a *t* -additive metric. The inherent difficulty in reconstructing phylogenies using additive SR functions is that computing the implied *t* -additive metric requires the *exact* values of the inter-taxon transition matrices {**P**_*ij*_}, and getting these exact values from alignments of finite length is practically impossible. Therefore, a distance-based reconstruction algorithm is useful in a realistic setting only if it has some robustness to error in distance estimation. In [25], Atteson observed that the topology of a phylogenetic tree *t* can be accurately (and efficiently) reconstructed from any dissimilarity mapping *d* which is sufficiently close to a *t* -additive metric, using certain “robust” distance-based algorithms^c^. Formally, “sufficiently close” is defined by the following relation:

#### Definition 2.3 (Near-additive mapping)

A dissimilarity mapping *d* on *L* (*t* ) is said to be *near-additive* w.r.t. *t* iff there exists a *t* -additive mapping ^*d* ⋆^ s.t.

(6) $$\begin{array}{c} \;||D,D^{⋆}|{|}_{\infty }\;\left(\overset{△}{=}\underset{\left\{i,j\}\subset L(T\right)}{\max}\{|D(i,j)\;-\;D^{⋆}(i,j)|\}\right)\;<\;\frac{1}{2}w_{\min}(D^{⋆})\;, \end{array}$$

where $w_{\min}(D^{⋆})$ is the minimal weight assigned to an internal edge^d^ by the edge weighting function corresponding to the additive metric ^*d* ⋆^.

For our results we will be using a generalization of this criterion, in which the mapping *d*^⋆^ can be any *affine-additive* mapping, defined below.

#### Definition 2.4 (Affine-additive mapping)

A dissimilarity mapping $D^{\prime }$ is said to be *affine-additive* w.r.t. a phylogenetic tree *t*, if there is a *t* -additive metric *d*, and scalars *a* >0,*B* s.t. $D^{\prime }=\mathrm{aD}+b$ (i.e., $D^{\prime }(i,j)=\mathrm{aD}(i,j)+b$ for all {i,j}⊂L(T)).

As with additive metrics, affine-additive mapping are also associated with edge weights. Let *d* be a *t* -additive mapping corresponding to the edge-weighting function *w* (·). Then the edge weighting function $w^{\prime }(\cdot )$ corresponding to the affine additive mapping $D^{\prime }=\mathrm{aD}+b$ is given by: $w^{\prime }(e)=\mathrm{aw}(e)$ for all internal edges, and $w^{\prime }(e)=\mathrm{aw}(e)+\frac{1}{2}b$ for all external edges. When *B* is positive, $D^{\prime }$ is actually an additive metric, but when *B* is negative, the weights of external edges implied by $w^{\prime }(\cdot )$ might be negative, and $D^{\prime }$ might even yield negative dissimilarities. The generalization of Atteson’s theorem to cases where *d*^⋆^ is affine-additive follows from the observation that the robust distance-based reconstruct algorithms considered by Atteson are invariant to affine transformations of their input distances. From this point on, when we say a dissimilarity mapping *d* is *near additive*, we mean it satisfies (6) with respect to some affine-additive mapping *d*^⋆^.

### Local consistency

Atteson’s result plays a central role in arguing the statistical consistency of distance-based phylogenetic reconstruction. Typically, this is done by assuming that the inter-leaf distances are computed using an SR function *Δ* which is additive for the underlying substitution model ℳ, as follows: 

1. If *Δ* is additive for ℳ, then for each ℳ-tree *t* the mapping $D_{\Delta }^{T}$ defined by $D_{\Delta }^{T}(i,j)=\Delta ({\text{P}}_{\mathrm{ij}})$ for all *i*,*j* ∈*L* (*t* ), is a *t* -additive metric.

2. As the length of the input sequences grows, the estimated transition matrices $\left\{{\hat{\text{P}}}_{\mathrm{ij}}\right\}$ converge (w.h.p.) to the true matrices {**P**_*ij*_}.

3. When $\left\{{\hat{\text{P}}}_{\mathrm{ij}}\right\}$ are sufficiently close to {**P**_*ij*_}, the estimated dissimilarity map $\hat{D}$ defined by $\hat{D}(i,j)=\Delta ({\hat{\text{P}}}_{\mathrm{ij}})$ is sufficiently close to $D_{\Delta }^{T}$, and is thus near-additive.

4. The near-additivity of the estimated dissimilarity map $\hat{D}$ implies accurate topological reconstruction, assuming a robust distance-based algorithm is used.

This line of argument has been used in numerous works studying statistical consistency of distance-based algorithms (e.g., [25-27]), and in all these cases an additive SR function is assumed. Notice, however, that this line of argument remains valid when $D_{\Delta }^{T}$ is *near additive* w.r.t. *t* . For instance, consistent reconstruction of any ℳ-tree is guaranteed by using an *affine-additive* SR function $\Delta^{\prime }$, which is an affine transformation of some additive SR function *Δ* : $\Delta^{\prime }=\mathrm{a\Delta}+b$ (with *a* >0). An SR function that is not affine-additive in a given substitution model ℳ does not guarantee consistency across all ℳ-trees, but it still can be consistent for specific ℳ-trees.

#### Definition 2.5 (Consistent SR function)

An SR function *Δ* of a substitution model ℳ is said to be *consistent* w.r.t. an ℳ-tree *t* if $D_{\Delta }^{T}$ is near-additive w.r.t *t* .

The main idea endorsed in this paper is that if an SR function only deviates slightly from some SR function which is affine-additive for ℳ, then it might be consistent with respect to many ℳ-trees of interest, and as such should be considered for use in distance based reconstructions.

## Deviation from additivity in homogeneous substitution models

In order to assess whether a given SR function *Δ* is consistent w.r.t. a given model tree *t*, one has to find an affine-additive mapping *d*^⋆^ which minimizes the ratio $\frac{||D_{\Delta }^{T},D^{⋆}|{|}_{\infty }}{w_{\min}(D^{⋆})}$ (see Definition 2.3). This task seems hard in a general setting, but in the special case of homogeneous substitution models it is tractable. Consider a homogeneous substitution model ${ℳ}_{R}$. The unit rate matrix **R**implies a 1-1 mapping between evolutionary time *t* and rate matrices in ${ℳ}_{R}$. It is thus useful to view an SR function for ${ℳ}_{R}$ as a function $\Delta :R^{+}\to R^{+}$ which maps the *evolutionary timet* to a dissimilarity measure *Δ* (*t* ).

It can be shown that such *Δ* is affine-additive in the model if and only if *Δ* (*t* )=*at* + *B* for some $a\in R^{+},b\in R$. We define the *deviation* of an SR function *Δ* from a given affine-additive function *at* + *B* in an interval [*t*_0_,*t*_1_] as $\frac{1}{a}\text{max}\{|\Delta (t)-\mathrm{at}-b|\;:t\in [t_{0},t_{1}]\}$ (the factor $\frac{1}{a}$ normalizes the deviation to units of evolutionary time). The *deviation from additivity* of *Δ* within [*t*_0_,*t*_1_] is defined as the minimum deviation of *Δ* from any affine-additive function in that interval.

### Definition 2.6 (Deviation from additivity)

Let $\Delta :R^{+}\to R^{+}$ be an SR function in a homogeneous substitution model. The *deviation from additivity* of *Δ* in an interval [*t*_0_,*t*_1_] is defined by:

(7) $$\begin{array}{c} \;\mathrm{dev}(\Delta ,[t_{0},t_{1}])\overset{△}{=}\underset{a\in R^{+},b\in R}{\inf}\left\{\underset{t\in [t_{0},t_{1}]}{\max}\left\{\frac{\left|\Delta (t)-\mathrm{at}-b\right|}{a}\right\}\right\}. \end{array}$$

Lemma 2.7 below presents the basic relation between deviation from additivity and consistency. In Section Performance of Non affine-additive SR functions in quartet resolution we demonstrate the tightness of this relation.

### Lemma 2.7

Let ℳ be a homogeneous model, and let *t* be an ℳ-tree with edge lengths (measured in time units) denoted by {*t*_*e*_}. Let _*t*_min__=min{*t*_*e*_:*e* ∈*t* }, and assume that all inter-leaf distances in *t* fall within the interval [*t*_0_,*t*_1_]. Then any SR function *Δ* in ℳ for which $\mathrm{dev}(\Delta ,[t_{0},t_{1}])<\frac{1}{2}t_{\min}$ is consistent w.r.t. *t* .

### Proof

We need to show that $D_{\Delta }^{T}$ is near-additive w.r.t. *t* . Since $\mathrm{dev}(\Delta ,[t_{0},t_{1}])<\frac{1}{2}t_{\min}$, there are $a\in R^{+},b\in R$ which satisfy

$$\underset{t\in [t_{0},t_{1}]}{\max}\left\{\frac{\left|\Delta (t)-\mathrm{at}-b\right|}{a}\right\}<\frac{1}{2}t_{\min}.$$

For all *i*,*j* ∈*L* (*t* ), denote $t_{\mathrm{ij}}=\underset{e\in \mathrm{pat}h_{T}(i,j)}{\sum }t_{e}$, and let *d* be the dissimilarity map associated with evolutionary time: *d* (*i*,*j* )=*t*_*ij*_. Clearly, *d* is an additive metric, and the dissimilarity mapping $D^{\prime }=\mathrm{aD}+b$ is an affine-additive mapping. The internal-edge-weights associated with $D^{\prime }$ are given by $w^{\prime }(e)=\mathrm{at}(e)$ (see discussion following Definition 2.4), implying that $w_{\min}(D^{\prime })=at_{\min}$. We thus have:

$$\begin{array}{c} ||D^{\prime },D_{\Delta }^{T}|{|}_{\infty }\leq \;\underset{t\in [t_{0},t_{1}]}{\max}\left\{\left|\Delta (t)-\mathrm{at}-b\right|\right\}\\ \;\;\;\;\;<\frac{1}{2}at_{\min}\;=\;\frac{1}{2}w_{\min}(D^{\prime })\;. \end{array}$$

□

**Figure 1:**
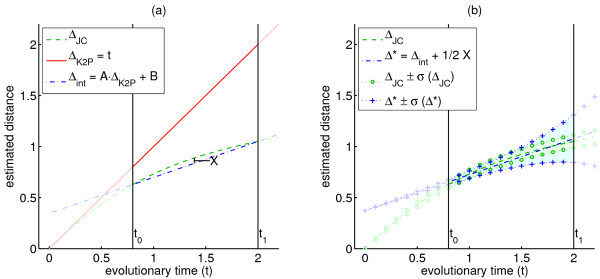
**Deviation from additivity and stochastic error. (a)*** Δ*_JC_ is portrayed (green) in the homogeneous sub-model of K2P with *R* =10 in the interval *t* ∈[0.8,2]. Its linear interpolation in that interval, *Δ*_int_=*At* + *B*, is plotted in blue, and the maximum difference between the two functions is designated by *X*. The deviation of *Δ*_JC_ from additivity in this setting is $\frac{X}{2A}$. **(b)** The affine-additive SR function minimizing its deviation from *Δ*_JC_ is $\Delta^{*}=\Delta_{\text{int}}+\frac{1}{2}X$. The two SR functions *Δ*_JC_ and *Δ*^∗^ are shown with their stochastic error margins, assuming sequence length of 500 bp.

An upper bound on the deviation of an SR function *Δ* from additivity in a given interval [*t*_0_,*t*_1_] is implied from the error associated with its linear interpolation *At* + *B* within that interval ( $A=\frac{\Delta (t_{1})-\Delta (t_{0})}{t_{1}-t_{0}}$ and $B=\frac{t_{1}\Delta (t_{0})-t_{0}\Delta (t_{1})}{t_{1}-t_{0}}$). Figure 1a demonstrates this for *Δ*_JC_ under a homogeneous sub-model of K2P, and Lemma 2.8 below presents a general upper bound on the deviation from additivity. For this purpose, we assume that the SR function *Δ* is a monotone increasing continuous function of *t* with continuous first and second derivatives.

### Lemma 2.8

Let $\Delta :R^{+}\to R^{+}$ be an SR function in a homogeneous substitution model, and let [*t*_0_,*t*_1_] be an interval. Let *Δ*_int_(*t* )=*At* + *B* be the linear interpolation of *Δ* in [*t*_0_,*t*_1_] defined above, and let $F\overset{△}{=}\max_{t\in [t_{0},t_{1}]}\{|\Delta^{\prime }(t)|\}$. Then

(8) $$\mathrm{dev}(\Delta ,[t_{0},t_{1}])\;\leq \;\frac{{\left(t_{1}-t_{0}\right)}^{2}F}{16A}.$$

### Proof

Let us start by introducing a couple of auxiliary notations:

$$\begin{array}{c} \psi (a,b,t)=\Delta (t)-\mathrm{at}-b\\ \psi (a,b)=\underset{t\in [t_{0},t_{1}]}{\max}\left\{\left|\psi (a,b,t)\right|\right\}.\;\;\;\; \end{array}$$

We are looking for $a\in R^{+}$ and b∈R which minimize $\frac{1}{a}\psi (a,b)$. Let _*ψ*_min__=_min*t* ∈[*t*_0_,*t*_1_]_{*ψ* (*A*,*B*,*t* )}, *ψ*_max_=_max*t* ∈[*t*_0_,*t*_1_]_{*ψ* (*A*,*B*,*t* )}, and let $b^{*}=B+\frac{1}{2}(\psi_{\text{max}}+\psi_{\min})$. Then $\psi (A,b^{*})=\frac{1}{2}(\psi_{\text{max}}-\psi_{\min})$. A bound for *dev* (*Δ*,[*t*_0_,*t*_1_]) will thus follow by showing that $\psi_{\text{max}}-\psi_{\min}\leq \frac{{\left(t_{1}-t_{0}\right)}^{2}F}{8}$.

Since *Δ*_int_(*t* )=*At* + *B* is a linear interpolation of *Δ* in *t*_0_*t*_1_, we have *ψ* (*ABt*_0_)=*ψ* (*ABt*_1_)=0. Let *t*_min_ be an arbitrary point in the interval *t*_0_*t*_1_s.t. *ψ* (*ABt*_min_)=*ψ*_min_≤0 and let (*t*_2_*t*_3_) be the maximal open interval in *t*_0_*t*_1_ containing *t*_min_ in which *ψ* (*ABt* )<0 (this interval can be empty if *ψ*_min_=0). We define a similar interval (*t*_4_*t*_5_) in which *ψ* (*ABt* )>0 around some arbitrary *t*_max_s.t. *ψ* (*ABt*_*max*_)=*ψ*_max_. Note that the intervals (*t*_2_*t*_3_) and (*t*_4_*t*_5_) are disjoint, and that *Δ*_int_ is the linear interpolation of *Δ* in both these intervals (since *ψ* (*ABt*_2_)=*ψ* (*ABt*_3_)=*ψ* (*ABt*_4_)=*ψ* (*ABt*_5_)=0). Therefore, the bound on the error of polynomial interpolation (see, e.g., [28], p. 187) implies that

$$\psi_{\min}\;\geq \;-\frac{{\left(t_{3}-t_{2}\right)}^{2}F}{8}\;\text{and}\;\psi_{\text{max}}\;\leq \;\frac{{\left(t_{5}-t_{4}\right)}^{2}F}{8}\;,$$

Combining these, we get

(9) $$\begin{array}{c} \mathrm{dev}(\Delta ,[t_{0},t_{1}])\leq \frac{1}{A}\psi (A,b^{*})=\frac{1}{2A}(\psi_{\text{max}}-\psi_{\min})\\ \;\leq \frac{\left({\left(t_{5}-t_{4}\right)}^{2}+{\left(t_{3}-t_{2}\right)}^{2}\right)F}{16A}\\ \leq \frac{{\left(t_{1}-t_{0}\right)}^{2}F}{16A}\;.\;\;\;\; \end{array}$$

□

### Note

In Appendix 3 we prove that if *Δ* does not intersect its linear interpolation *Δ*_int_=*At* + *B* within the interval (*t*_0_,*t*_1_), then the function *At* + *B*^∗^mentioned in the proof above is, in fact, the affine-additive function which minimizes the deviation from additivity of *Δ* in [*t*_0_,*t*_1_]. This means that, in such cases, the first inequality in (9) holds in equality. The last inequality in (9) also holds in equality in such cases, because we are guaranteed to have either [*t*_2_,*t*_3_]=[*t*_0_,*t*_1_] (when *Δ* is bounded from above by its linear interpolation) or [*t*_4_,*t*_5_]=[*t*_0_,*t*_1_] (when *Δ* is bounded from below by its linear interpolation). Thus, in such a case, the bound of Lemma 2.8 is reduced to the bound on interpolation error (middle inequality in (9)). Cases where *Δ* does not intersect its linear interpolation are frequent among many SR functions of interest, as this condition holds when *Δ* is either convex or concave.

### Deviation of *Δ*_JC_from Additivity in K2P

We now turn to study the deviation of *Δ*_JC_ from additivity in homogeneous sub-models of K2P with ti-tv ratio $R>\frac{1}{2}$. First, we express *Δ*_JC_as a function of the ti-tv ratio *R* and the time *t*, using (5) and the relations $\frac{\alpha }{2\beta }=R$ and *α* + 2*β* =1.

(10) $$\begin{array}{c} \Delta_{\mathrm{JC}}(R,t)=-\frac{3}{4}\text{ln}\;\left(\frac{1}{3}(e^{-4\mathrm{\beta t}}+2e^{-2\mathrm{\alpha t}-2\mathrm{\beta t}})\right)\\ \;=-\frac{3}{4}\text{ln}\;\left(\frac{1}{3}(e^{-\frac{2t}{R+1}}+2e^{-t\frac{2R+1}{R+1}})\right)\\ \;=-\frac{3}{4}\text{ln}\;\left(\frac{1}{3}e^{-\frac{2t}{R+1}}\left(1+2e^{t\frac{2R-1}{R+1}}\right)\right)\\ \;=\left(\frac{3}{2(R\;+\;1)}\right)t\;-\;\frac{3}{4}\text{ln}\;\left(\frac{1}{3}\left(1\;+\;2e^{-t\frac{2R-1}{R+1}}\right)\right). \end{array}$$

Note that the homogeneous K2P sub-model with $R=\frac{1}{2}$ is the JC model; in this case the second term of (10) vanishes, leaving $\Delta_{\mathrm{JC}}(\frac{1}{2},t)=t$. For other homogeneous sub-models of K2P, where $R>\frac{1}{2}$, *Δ*_JC_ is not affine-additive (i.e., not of the form *at* + *B* for *a* >0), and we can use the result in Lemma 2.8 to bound the deviation of *Δ*_JC_from additivity. Denoting $\rho =\frac{2R-1}{R+1}$, we get

(11) $$\begin{array}{ccc} \frac{\partial \Delta_{\mathrm{JC}}(R,t)}{\mathrm{\partial t}} & = & \frac{3}{2(R+1)}+\frac{3}{2}\rho \frac{e^{-\mathrm{\rho t}}}{1+2e^{-\mathrm{\rho t}}}\;>\;0\;. \end{array}$$

(12) $$\begin{array}{ccc} \frac{\partial^{2}\Delta_{\text{JC}}(R,t)}{\partial t^{2}} & = & -\frac{3}{2}\rho^{2}\frac{e^{-\mathrm{\rho t}}}{{\left(1+2e^{-\mathrm{\rho t}}\right)}^{2}}\;<\;0\;. \end{array}$$

(13) $$\begin{array}{ccc} \frac{\partial^{3}\Delta_{\text{JC}}(R,t)}{\partial t^{3}} & = & \frac{3}{2}\rho^{3}\frac{(1-2e^{-\mathrm{\rho t}})e^{-\mathrm{\rho t}}}{{\left(1+2e^{-\mathrm{\rho t}}\right)}^{3}}\;. \end{array}$$

We get that for any given ti-tv ratio $R>\frac{1}{2}$, *Δ*_JC_(*R*,*t* ) is a concave monotone increasing function, and its second derivative attains a global minimum of $-\frac{3}{16}\rho^{2}$ at $t=\frac{\text{ln}\;(2)}{\rho }$. By the note following Lemma 2.8, the deviation of *Δ*_JC_ from additivity in an interval [*t*_0_,*t*_1_] can be evaluated by computing the linear interpolation *Δ*_int_=*At* + *B* of *Δ*_JC_ in [*t*_0_,*t*_1_], and finding *t* ∈[*t*_0_,*t*_1_] which maximizes *Δ*_JC_(*t* )−*Δ*_int_(*t* ) (see Figure 1a). A bound on this deviation from additivity can be obtained through Lemma 2.8 by plugging in the slope of the linear interpolation, *A*, and the maximum value, *F*, attained by the second derivative of *Δ*_JC_ in [*t*_0_,*t*_1_]. Using Lemma 2.7 and an expression for *dev* (*Δ*_JC_(*R*,*t* ),[*t*_0_,*t*_1_]), it is possible to map out coherent collections of homogeneous K2P-trees for which *Δ*_JC_ is guaranteed to be consistent. Each collection is defined by a range of ti-tv ratios [0.5,*R*_max_], a range of inter-leaf distances [*t*_0_,*t*_1_], and a lower bound on the weights of internal edges in the tree, given by *t*_min_=2*dev* (*Δ*_JC_(*R*_max_,*t* ),[*t*_0_,*t*_1_]).

After determining a collection of trees for which a given non-affine-additive SR function, *Δ*, is consistent, one can compare the performance of *Δ* with additive alternatives. In our case, we compare *Δ* =*Δ*_JC_, which is not affine additive when $R>\frac{1}{2}$, to the standard additive SR function *Δ*_K2P_. The potential advantage of *Δ*_JC_over *Δ*_K2P_ lies in its reduced *stochastic noise* . Informally, this occurs because JC relies on the accuracy of estimating a single parameter - the sum *p* =*p*_*α*_ + 2*p*_*β*_, while *Δ*_K2P_ relies on the accuracy of estimating each of the two parameters *p*_*α*_and *p*_*β*_ separately. The stochastic noise of an SR function is measured by the *standard deviation* of the statistical estimator associated with it, denoted *σ* (*Δ*_JC_) and *σ* (*Δ*_K2P_), respectively. We use the result in [9] to get a first order approximation (based on the delta method [29]) of *σ* (*Δ*_K2P_) for sequences of length *k* and model parameters *Rt* :

(14) $$\begin{array}{c} \sigma (\Delta_{\text{K2P}})\\ \approx \sqrt{\frac{\left(e^{\frac{4t}{R+1}}-1)\;+\;4(e^{\frac{2t}{R+1}}\;-\;1)\;\;+\;2(e^{\frac{4\mathrm{Rt}}{R+1}}(e^{\frac{4t}{R+1}}+1)-2\right)}{16k}}\;. \end{array}$$

By a similar application of the delta method to *Δ*_JC_, we obtain:

(15) $$\sigma (\Delta_{\text{JC}})\approx \sqrt{\frac{p(t,R)\;(1-p(t,R))}{k\;{\left(1-\frac{4}{3}p(t,R)\right)}^{2}}}\;,$$

where *k* is the sequence length and $p(t,R)=p_{\alpha }+2p_{\beta }=\frac{3}{4}-\frac{1}{4}e^{-\frac{2t}{R+1}}-\frac{1}{2}e^{-\frac{(2R+1)t}{R+1}}$ (see (3)).

Figure 1 provides an illustrative comparison of *Δ*_JC_and *Δ*_K2P_ under the homogeneous sub-model of K2P with ti-tv ratio *R* =10, and within the inter-leaf time interval of [0.8,2]. Figure 1a shows the deviation of *Δ*_JC_from additivity in that setting, using its linear interpolation *Δ*_int_=*At* + *B* . Note that Lemma 2.8 and the subsequent note imply that $\mathrm{dev}(\Delta_{\text{JC}},[0.8,2])=\frac{X}{2A}$, where *X* =max*t*_∈[0.8,2]_{*Δ*_JC_(*t* )−*Δ*_int_(*t* )}. Figure 1b depicts *Δ*_JC_ in the same setting with its stochastic error margins (*Δ*_JC_±*σ* (*Δ*_JC_)), alongside its closest affine-additive function $\Delta^{*}=\Delta_{\text{int}}+\frac{1}{2}X$ and its stochastic error margins (*Δ*^∗^±*σ* (*Δ*^∗^)). These stochastic error margins are determined by assuming a sequence length of 500 bp in the first-order approximations given in (14) and (15), where *σ* (*Δ*^∗^) is given by scaling *σ* (*Δ*_K2P_) by the slope *A* of the linear interpolation. Note how the margins of *Δ*_JC_are actually more tightly concentrated around its affine-additive approximation *Δ*^∗^ than the margins of *Δ*^∗^. This implies that, despite its deviation from additivity in this setting, distances obtained using *Δ*_JC_are actually more likely to be near-additive than distances obtained using *Δ*_K2P_.

## Performance of Non affine-additive SR functions in quartet resolution

The quartet tree is the smallest phylogenetic tree with non-trivial topology. Focusing on quartets enables a close study of the effects of deviation from additivity and stochastic noise on reconstruction accuracy. The topology of a quartet spanning four taxa {1,2,3,4} can be represented by the split notation (*ij* |*kl* ) (where {*ijkl* }={1,2,3,4}), indicating that the internal edge of the quartet separates *ij* from *kl* . All distance based quartet resolution algorithms essentially reduce to the four-point method (FPM) [26,30], which resolves this split using the six observed pairwise distances $\left\{{\hat{d}}_{\mathrm{ij}}:\{i,j\}\subset \{1,2,3,4\}\right\}$: it first partitions the six observed distances into three sums ${\hat{d}}_{12}+{\hat{d}}_{34}$, ${\hat{d}}_{13}+{\hat{d}}_{24}$, and ${\hat{d}}_{14}+{\hat{d}}_{23}$, and then determines the quartet split according to the minimal sum (the sum ${\hat{d}}_{\mathrm{ij}}+{\hat{d}}_{\mathrm{kl}}$ corresponds to the split (*ij* |*kl* )). We will focus on the task of reconstructing homogeneous K2P quartets using FPM with distances $\left\{{\hat{d}}_{\mathrm{ij}}\right\}$ estimated using either *Δ*_JC_or *Δ*_K2P_. We note that most of our findings easily generalize to more sophisticated homogeneous substitution models, replacing *Δ*_JC_ by any concave distance function and *Δ*_K2P_ by some SR function corresponding to the evolutionary time *t* .

**Figure 2:**
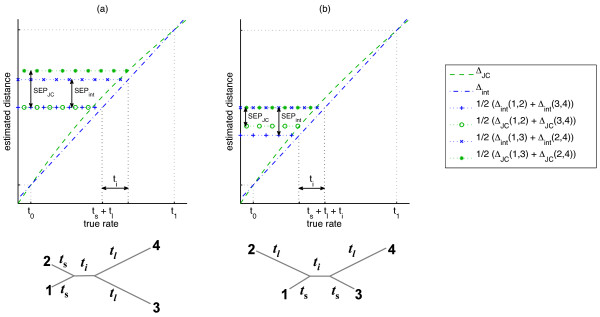
**Performance of the Four Point Method using *Δ*_JC_ on K2P quartets with ti-tv ratio *R* = 2**. The concave non affine-additive SR function *Δ*_JC_ is shown (dashed green line) in the interval [*t*_0_,*t*_1_], where *t*_0_ and *t*_1_ are the smallest and largest of the six pairwise distances (resp.). The dashed blue line shows the linear interpolation *Δ*_int_=*At* + *B* of *Δ*_JC_ in the interval [*t*_0_,*t*_1_]. Horizontal dotted lines correspond to half of the two competing sums computed by FPM under the two SR functions (see legend). **(a)** In quartets of type A, *t*_0_=*t*_12_ and *t*_1_=*t*_34_, and so *Δ*_int_(1,2) + *Δ*_int_(3,4)=*Δ*_JC_(1,2) + *Δ*_JC_(3,4). However, for *i* ∈{1,2} and *j* ∈{3,4}, *Δ*_int_(*i*,*j* )<*Δ*_JC_(*i*,*j* ). Therefore, the deviation from additivity of *Δ*_JC_* increases* its FPM separation, denoted _SEPJC_, compared to the FPM separation _SEPint_ of *Δ*_int_. **(b)** In quartets of type B, *t*_0_=*t*_13_ and *t*_1_=*t*_24_, and so *Δ*_int_(1,3) + *Δ*_int_(2,4)=*Δ*_JC_(1,3) + *Δ*_JC_(2,4). However, *Δ*_int_(1,2)=*Δ*_int_(3,4)<*Δ*_JC_(1,2)=*Δ*_JC_(3,4), and so *Δ*_int_(1,2) + *Δ*_int_(3,4)<*Δ*_JC_(1,2) + *Δ*_JC_(3,4). Therefore, the deviation from additivity of *Δ*_JC_* decreases* its FPM separation, denoted _SEPJC_, compared to the FPM separation _SEPint_ of *Δ*_int_. Note that _SEPint_ remains invariant in both types of quartets under fixed *t*_*i*_ whereas _SEPJC_ changes, depending on the type of quartet and the *t*_*s*_/*t*_*l *_ratio.

For concreteness, we assume henceforth that the quartet split is (12|34), meaning that the sum of the exact evolutionary times *t*_12_ + *t*_34_ is minimal. We start by analyzing the impact of the deviation from additivity of *Δ*_JC_ on the consistency of quartet resolutions. First, observe that *any* monotone distance function is consistent for quartets in which *t*_12_ and *t*_34_ are the smallest interleaf distances - as is the case with symmetric quartets, in which all external edges are of the same length. Therefore, we study two prototypes of asymmetric quartets. The length of the internal edge in both types is *t*_*i*_, and each type has two long external edges of length *t*_*l*_, and two short external edges of length *t*_*s*_. In type A quartets (Figure 2a), the short edges are on one side of the split and the long edges are on the other side. In this case *d*_12_and *d*_34_ re the smallest and largest interleaf distances (resp.). Hence, the concavity of *Δ*_JC_ increases the separation between the sum *d*_12_ + *d*_34_ and the other two competing sums, leading to an expected *improvement* in reconstruction accuracy. The other quartet configuration (type B; Figure 2b) has a short edge and a long edge on both sides of the split. In this case, the interval of interpolation is [*d*_13_,*d*_24_], and the distance *d*_12_=*d*_34_ is near the center of this interval. Thus the concavity of *Δ*_JC_ decreases the separation between the sums *d*_13_ + *d*_24_ and *d*_12_ + *d*_34_ by approximately twice the deviation from additivity of *Δ*_JC_ in that range.

When the deviation from additivity exceeds half the length of the internal edge, the sum *d*_13_ + *d*_24_ becomes the minimal sum, and *Δ*_JC_ becomes inconsistent. Note that this demonstrates the tightness of the condition stated in Lemma 2.7, and in this sense, type B quartets provide a worst case scenario for quartet resolution by a concave SR function^e^.

**Figure 3:**
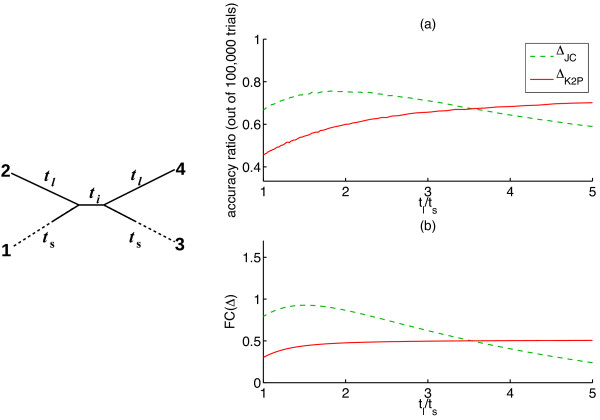
**Performance of *Δ*_*JC*_ and *Δ*_*K*2*P*_ on a series of quartets of type B.** A series of homogeneous K2P quartets is considered (left illustration), with ti-tv ratio of *R* =5, and edge lengths *t*_*i*_=0.2, *t*_*l*_=1, and *t*_*s*_∈[0.2,1]. **(a)** Reconstruction accuracy using FPM and either *Δ*_JC_ (dashed green) or *Δ*_K2P_ (solid red) plotted against *t*_*l*_/*t*_*s*_. Accuracy ratio is estimated using 100,000 independent replicates for each value of *t*_*s*_ in the interval [0.2,1] (in steps of 0.01), with sequence length 1,000 bp. **(b)** Fisher’s Criterion (FC) for the sums corresponding to splits (12|34) and (13|24) under either *Δ*_JC_ (dashed green) or *Δ*_K2P_ (solid red) plotted against *t*_*l*_/*t*_*s*_.

Next we turn to compare the accuracy of *Δ*_JC_ with that of *Δ*_K2P_ when used to reconstruct its “worst case scenario” quartets of type B. Interestingly, *Δ*_JC_ ends up outperforming *Δ*_K2P_ on many of these quartets, due to its reduced stochastic noise (as predicted in our discussion revolving around Figure 1b). For example, consider a series of homogeneous K2P quartets of type B with ti-tv ratio *R* =5, whose edge lengths were set as follows: *t*_*i*_=0.2, *t*_*l*_=1.0, and *t*_*s*_∈[0.2,1.0]. We assessed reconstruction accuracy for both SR functions (*Δ*_JC_and *Δ*_K2P_) across this series of quartets, by generating 100,000 simulations of the substitution process using 1,000 bp long sequences for each quartet (Figure 3a). Despite its deviation from additivity, *Δ*_JC_ outperforms the additive SR function *Δ*_K2P_ on many of these quartets (as long as *t*_*l*_/*t*_*s*_<3.6) . Note that as *t*_*s*_ shrinks, the deviation of *Δ*_JC_ from additivity increases, since the interval [*t*_0_,*t*_1_] expands. This experiment appears to indicate that the deviation of *Δ*_JC_ from additivity has to be quite large for *Δ*_K2P _ to outperform it.

### Fisher’s criterion for separability

We now present a simple and general framework based on the so-called Fisher Criterion (FC) for predicting the relative accuracy of two SR functions in resolving quartets. FC measures the effective separation between normal random variables *X* ∼*n* (*μ*_1_*σ*_1_) and *Y* ∼*n* (*μ*_2_*σ*_2_) using the following measure^f^ ( [20,21]):

(16) $$\mathrm{FC}(X,Y)=\frac{\left|\mu_{1}-\mu_{2}\right|}{\sqrt{\sigma_{1}^{2}+\sigma_{2}^{2}}}\;.$$

We use FC to measure the separability of the distance sum corresponding to the true split (which should be the minimal sum for consistent SR functions) from the two remaining sums. For the expectation *μ* of each sum we use the true distances as computed by the SR function on the actual model parameters. For the variance ^*σ* 2^, we use the sum of the approximate variances of the two distances involved in the sum. We expect that an SR function which provides a larger separation of the smallest sum from the two other sums will imply a better reconstruction probability.

We note that FC is not an exact indicator of the separability in our case, because the necessary criteria for this are not satisfied in our model. Namely, the two distance sums are not normally distributed, and they are correlated through the substitution process along the external edges of the quartet. Nevertheless, as Figure 3b suggests, FC turns out to provide a quite reliable comparison of the expected performance of *Δ*_JC_ and *Δ*_K2P_ for the quartet series considered in the aforementioned experiment. Figure 3b exhibits for each quartet the FC of *Δ*_JC_ alongside that of *Δ*_K2P_, both associated with the comparison of the true split (12|34) and the “*Δ*_JC_ favored split” (13|24). As shown, the trends observed in both FC plots closely resemble the trends observed in the reconstruction accuracy plot (Figure 3a), and the the equilibrium point of the FC values of *Δ*_JC_ and *Δ*_K2P_ is very close to the equilibrium point of the accuracy of reconstructions of these two functions (near *t*_*l*_/*t*_*s*_=3.6).

A useful feature of this framework is the natural way in which it teases apart the stochastic noise from the deviation from additivity. If we denote the numerator of FC by *SEP* (for “separation”) and its denominator by *NOISE*, then a comparison of FC estimates between two SR function *Δ*_1_,*Δ*_2_ can be represented as a ratio of ratios:

(17) $$\frac{\mathrm{FC}(\Delta_{1})}{\mathrm{FC}(\Delta_{2})}=\frac{\mathrm{SEP}(\Delta_{1})}{\mathrm{SEP}(\Delta_{2})}/\frac{\mathrm{NOISE}(\Delta_{1})}{\mathrm{NOISE}(\Delta_{2})}\;.$$

**Figure 4:**
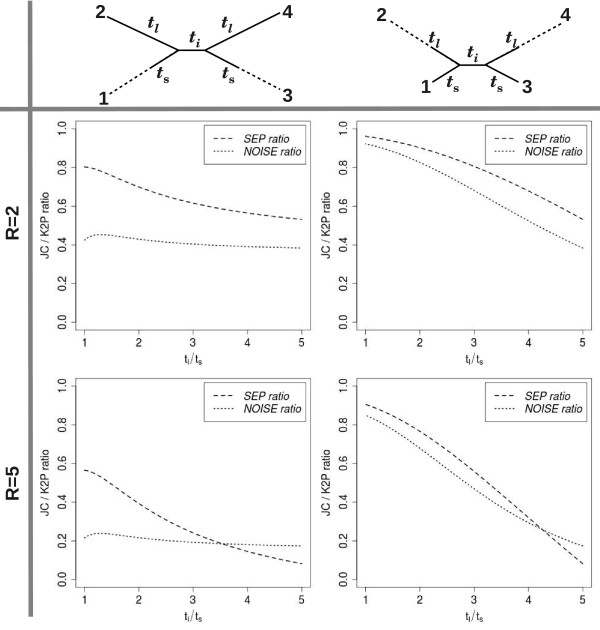
*** SEP* and *NOISE* ratios.***SEP* (*Δ*_JC_)/*SEP* (*Δ*_K2P_) (dashed) and *NOISE* (*Δ*_JC_)/*NOISE* (*Δ*_K2P_) (dotted) plotted against *t*_*l*_/*t*_*s*_ for four series of homogeneous K2P quartets of type B. Top two series have ti-tv ratio of *R* =2, and bottom two series have ti-tv ratio of *R* =5. Left two series have external edge lengths *t*_*l*_=1 and *t*_*s*_∈[0.2,1], and right two series have external edge lengths *t*_*l*_∈[0.2,1] and *t*_*s*_=0.2. The length of the internal edge is constant *t*_*i*_=0.2 in all four series.

Figure 4 illustrates how a comparison between the expected performance of *Δ*_JC_ and that of *Δ*_K2P_ can be carried out by tracing the *SEP* and *NOISE* ratios along four series of homogeneous K2P quartet: the bottom-left plot corresponds to the quartet series considered in Figure 3; the plot above it corresponds to the same series with ti-tv ratio *R* =2; the two plots on the right describe two quartet series in which the weight of the short edges is constant *t*_*s*_=0.2, and the weight of the long edges ranges in [0.2,1]. These four series demonstrate several typical trends in the behavior of the *SEP* and *NOISE* ratios. First, we observe that the *NOISE* ratio decreases (favoring *Δ*_JC_) as the diameter of the quartet (*t*_24_) increases (it is almost constant in the two series on the left, and monotone decreasing in the series on the right). This is because the diameter provides the major contribution to the stochastic noise (for both *Δ*_JC_ and *Δ*_K2P_), and as it increases, the ratio between the stochastic noise of *Δ*_K2P_ and *Δ*_JC_ increases as well. We also observe a natural decrease in the *NOISE* ratio with an increase in the ti-tv ratio (the *NOISE* ratio for *R* =5 is consistently smaller than for *R* =2). Concerning the *SEP* ratio, we see it becomes smaller (favoring *Δ*_K2P_) as the quartet becomes more unbalanced (the *SEP* ratio decreases along the X axis in each of the four plots). This is because the deviation of *Δ*_JC_ from additivity increases as the inter-leaf distance interval [*t*_0_,*t*_1_]=[*t*_13_,*t*_24_] expands. Deviation of *Δ*_JC_ from additivity also increases with the ti-tv ratio, as the substitution model further departs from the assumptions of JC (the *SEP* ratio for *R* =5 is consistently smaller than for *R* =2).

The two series on the right side of Figure 4 demonstrate well the tradeoff between the effects of stochastic noise and deviation from additivity. In both series, the *SEP* and *NOISE* ratios decrease as the quartets become more unbalanced (due to the trends listed above). However, the rates of decrease of these two ratios are different due to the different ti-tv ratios, and this determines the expected relative performance of the two SR functions across the series. When * R * = 2, the *SEP* ratio decreases at a slower rate than the *NOISE* ratio, and *Δ*_JC_ is expected to outperform *Δ*_K2P_ across the entire series. When * R * = 5, the *SEP* ratio decreases at a faster rate than the *NOISE* ratio, and when the quartets are sufficiently unbalanced (*t*_*l*_/*t*_*s*_>4) *Δ*_K2P_ is expected to outperform *Δ*_JC_.

## Simulations on Hasegawa’s Tree

**Figure 5:**
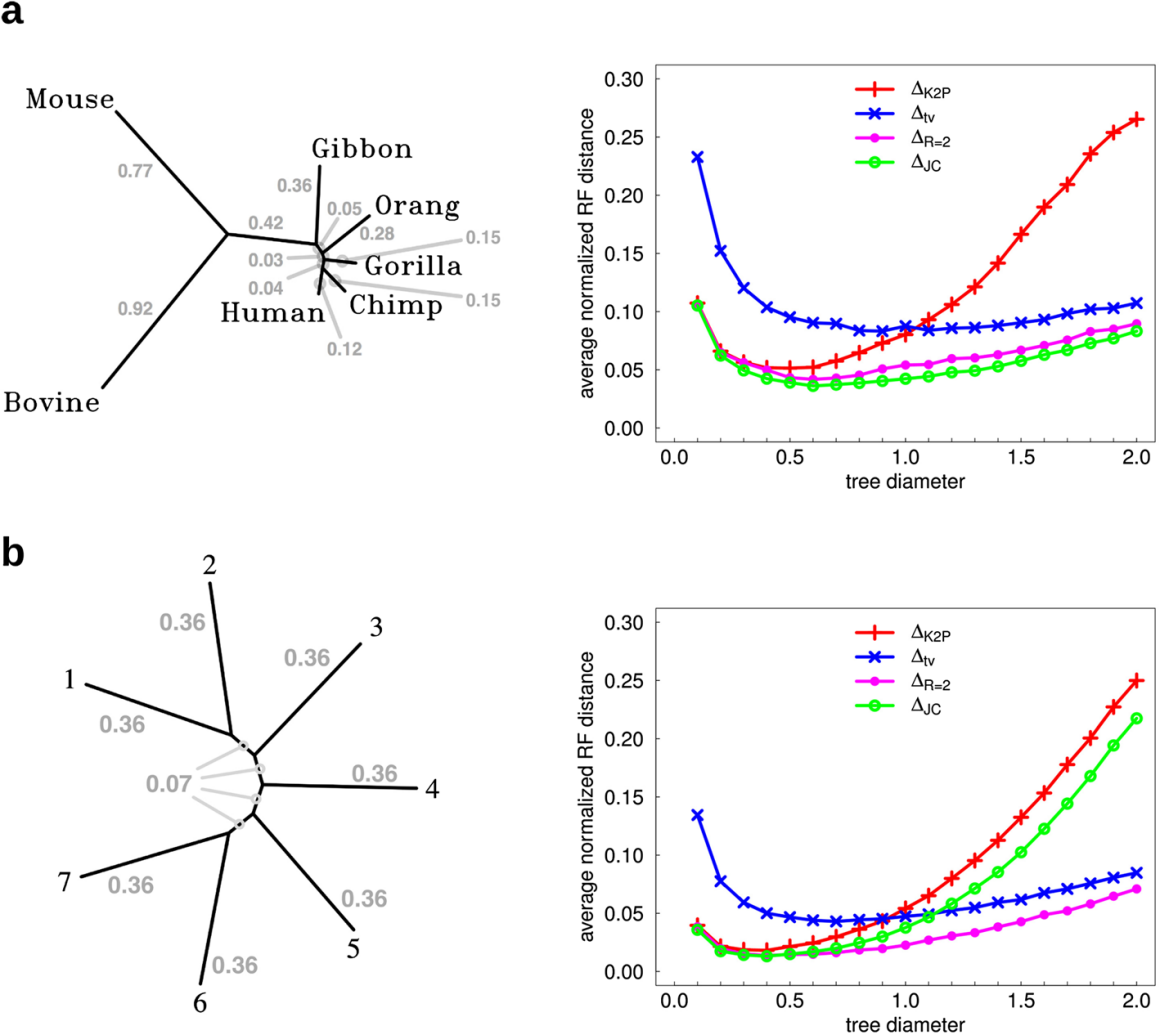
**Simulations on Hasegawa’s Tree. (a)** Reconstruction accuracy of four different SR functions on different scaled versions of Hasegawa’s tree [6]. The tree with scaled edge weights is depicted (left) next to the graph (right) plotting reconstruction accuracy of four SR functions. Different scales of the tree are considered, indicated by the diameter of the tree (X axis). Reconstruction accuracy (Y axis) is measured for each scaled tree by the average normalized RF distance between the reconstructed tree and the true tree across 10,000 simulated data sets. Simulations were carried out assuming a ti=tv ratio of *R* = 2 and sequence length of 500 bp. **(b)** A similar plot is shown for a semi-symmetric caterpillar tree.

In this section we describe experiments done on simulated data sets generated along the seven-taxon tree assembled by Hasegawa, Kishino, and Yano in 1985 [1,6]. This tree, spanning seven eutherian mammals (Figure 5a), was reconstructed originally using mitochondrial DNA sequences. It has a caterpillar topology (meaning that every internal node is incident to an external edge), and it has long external edges and short internal edges, making it a suitable representative of small phylogenetic trees spanning moderately distant species. These features also make it particularly challenging for distance-based reconstruction.

In our study we used the tree structure and edge lengths to generate simulated data sets. We considered the tree in various scales, by setting the tree diameter (largest inter-taxon path length) to values in the interval [0.1,2.0]. For each scale considered, 10,000 simulations were carried out, where in each simulation 500 bp sequences were evolved along the tree according to a homogeneous K2P substitution model with ti-tv ratio of *R* =2. For each simulated data set, estimated values of the K2P statistics *p*_*α *_and *p*_*β*_, denoted by ${\hat{p}}_{\alpha }$ and ${\hat{p}}_{\beta }$, were extracted for all 72 pairs of taxa. Subsequently, several distance matrices were computed for each data set by applying different SR functions to these estimated statistics. Reconstruction accuracy was evaluated by applying the Neighbor Joining (NJ) algorithm [31,32] to these distance matrices and recording the *Robinson-Foulds topological distance* (RF) [33] between the reconstructed tree and the Hasegawa tree. Sequence simulation was performed using SeqGen [34] (by choosing the HKY model with uniform base frequencies), and tree reconstruction was performed using the version of NJ implemented in the PHYLIP package [35].

We studied the reconstruction accuracy associated with four different SR functions: *Δ*_JC_, *Δ*_K2P_, *Δ*_tv_, and *Δ*_R=2_. The first two are as described in Equations (5) and (4), respectively. The third SR function, *Δ*_tv_, considers only tv-type substitutions: $\Delta_{\mathrm{tv}}(p_{\alpha },p_{\beta })\;=\;-\frac{1}{4}\text{log}\;\left(1-4p_{\beta }(t)\right)\;=\;\mathrm{\beta t}$, and the fourth SR function, *Δ*_R=2_, is based on a maximum likelihood (ML) estimator^g^ of the time *t* from the estimated transition probabilities ${\hat{p}}_{\alpha },{\hat{p}}_{\beta }$, given that *R* =2. Informally, this function, which uses knowledge of the true value of *R* (which is typically unknown to the user), is optimal in our setting, because it has similar stochastic noise as *Δ*_JC_, and it is additive since it coincides with *Δ*_K2P_ when applied to transition probabilities ${\hat{p}}_{\alpha },{\hat{p}}_{\beta }$ that are consistent with a ti-tv ratio of *R* =2.

The performance of these four SR functions is traced across the different tree scales in Figure 5a. For each SR function *Δ* and scale *s*, we recorded the average normalized RF distance from the true tree to each of the 10,000 trees reconstructed using *Δ* . The RF distance was normalized by its maximum value which is twice the number of internal edges in the tree (in our case 2×4=8). As observed previously in [9], *Δ*_K2P_ performed well in shorter scales, and *Δ*_tv_ performed well in longer scales. However, both additive SR functions were significantly outperformed in nearly all cases by *Δ*_JC_. Surprisingly, *Δ*_JC_ even slightly outperformed *Δ*_R=2_. We speculate that this happened due to a bias similar to the one observed in type A quartets in Section Performance of Non affine-additive SR functions in quartet resolution, improving the performance of concave SR functions such as *Δ*_JC_ on certain K2P-trees.

To test this hypothesis, we went through a similar experiment with a more symmetric seven-taxon caterpillar tree, with internal edges of uniform length *t*_*int*_, and external edges of uniform length *t*_*ext*_=5*t*_*int *_(Figure 5b). The symmetry of this tree was expected to reduce the effect of the reconstruction bias observed in Hasegawa’s tree, and indeed, *Δ*_JC_ performed much more poorly on this tree. Despite this fact, *Δ*_JC_ still outperformed *Δ*_K2P_ in all scales and *Δ*_tv_ in the smaller scales (*s* <1.1).

## Inferring trees from genomic sequences

In this section we describe our study comparing various SR functions on genomic DNA sequences. Next to *Δ*_JC_ and *Δ*_K2P_ we also considered the well known LogDet SR function [36,37], denoted here as *Δ*_LogDet_. Extending our study to this setting is challenging in two respects. First of all, unlike the simulated case, the true tree is not known with complete confidence, and accuracy of reconstruction can only be determined by using a well-accepted reference tree that may contain some errors. Secondly, the true substitution model is also unknown and is likely to violate the assumptions of both JC and K2P models and even the relaxed assumptions of the general time-reversible model (in which *Δ*_LogDet_ is additive). Hence, we have to assume in this case that *Δ*_JC_, *Δ*_K2P_, and *Δ*_LogDet_ are all non affine-additive, where *Δ*_JC_ and *Δ*_K2P_ are still likely to exhibit higher deviation from additivity than *Δ*_LogDet_, since they make stronger assumptions on the substitution model.

### The genomic data set

In building the genomic data set, we made use of a set of 31 *clusters of orthologous groups* (COGs) which was compiled by Ciccarelli et al. and used for inferring phylogenetic relationships amongst a large number of species in [38,39]. These 31 gene families were selected to capture the evolutionary history of the species containing them. This was done in [38] by making sure that the genes in these families have the following properties: (1) they are highly conserved across species, (2) they have a small number of paralogs, and (3) they are weakly affected by horizontal gene transfer. We scanned the NCBI genome database and found 199 bacterial genomes that contained all annotated COGs. For each of the 31 COGs, we extracted the appropriate protein sequence in each of the 199 bacterial species, choosing an arbitrary paralog in cases of multiple hits. We followed a procedure similar to the one described in [38,39] to obtain reliable multiple-sequence alignments for each COG: we computed a 199-way multiple alignment of the protein sequences of each COG using HMMalign [40] and then mapped each protein sequence back to its coding DNA sequence. The conserved parts of each of the 31 DNA alignments were extracted using GBLOCKS [41] to filter out alignment columns with 50% or more gap symbols. The alignments were manually scanned, and 36 species which contributed a large number of gaps to the alignments were removed from the subsequent analysis. The 31 different alignments were concatenated to form one long 163-way multiple sequence DNA alignment.

For the reference tree we used the phylogenetic tree of microbial species provided by the ARB-SILVA Living Tree Project [42]. This tree, spanning 8,029 species at the time of writing, is based on a widely accepted analysis of the small subunit (SSU) 16S RNA. A subtree spanning our 163 bacterial species was extracted from this tree and treated as the true phylogenetic tree in our analysis.

### Reconstruction accuracy for ten-species subsets

We used the base set of 163 species to generate 40,000 random 10-species sub-alignments. The random selection process was guided to generate species subsets corresponding to a wide range of diameter scales (a blind random selection process is biased toward subsets with large diameters). For each of the 40,000 subsets, a 10-way subalignment was extracted from the original 163-way alignment, and in this alignment we extracted only columns corresponding to four-fold degenerate sites that do not have any gap symbol. This is done to make sure the sites used for distance estimation have undergone a substitution process that is as uniform as possible along the different lineages and across the different sites. Each sub-alignment was used to compute three distance matrices – one under *Δ*_JC_, one under *Δ*_K2P_, and one under *Δ*_LogDet_. The latter was calculated by the version that is implemented in the PHYLIP package. The NJ algorithm was then applied to the three matrices and the resulting trees were compared to the true tree (as depicted by the appropriate LTP subtree) according to the RF distance.

As an additional comparison, we used a fourth reconstruction technique. This method (termed BIONJ-GTR) used the BIONJ reconstruction algorithm [43] on distances obtained under the general time-reversible model with invariant sites and Gamma distribution of rates across variant sites (GTR+*Γ* +I) [8,44].

**Figure 6:**
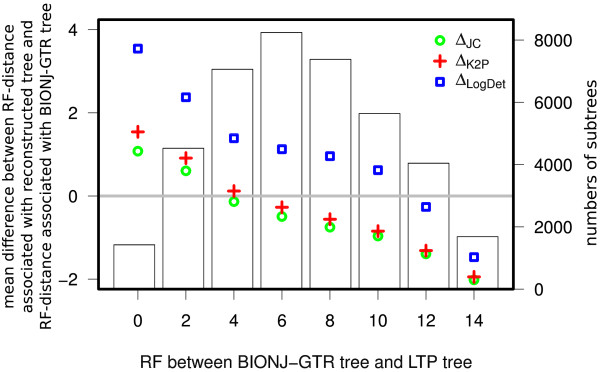
**Evaluation against BIONJ-GTR tree.** The 40,000 subsets of size 10 were partitioned according to the the RF-distance between the reference LTP tree and the tree reconstructed using BIONJ-GTR (X axis). The (left) Y axis describes the mean difference between the RF-distance associated with a tree reconstructed using a particular SR function (*Δ*_K2P_, *Δ*_JC_, or *Δ*_LogDet_) and the RF-distance associated with the BIONJ-GTR tree. The bar plot in the background depicts the number of subsets in each bin.

The PhyML package [45] was used to infer this tree for each of the 40,000 subsets. We selected the GTR+*Γ* +I model since it was found by the MEGA5 software [46] to provide the best fit to the sequence data. The 40,000 sampled instances were partitioned into eight bins according to the RF distance observed between the BIONJ-GTR tree and the true (LTP) tree, and average RF distances were recorded for each of the three SR functions in each bin. This allowed us to observe trends throughout these 40,000 samples (Figure 6). Of the 40,000 trees inferred under *Δ*_JC_, 83.1% showed an equal or lower RF distance than those reconstructed by the BIONJ-GTR method. Moreover, *Δ*_JC_ outperformed *Δ*_K2P_ and *Δ*_LogDet_ on average in all partitions, and *Δ*_LogDet_ showed by far the worst performance with 48.7% of all reconstructed trees achieving higher RF distances to the reference tree than those inferred by BIONJ-GTR. As with our results on simulated data sets, we see that the SR functions with lower stochastic error but inferior model fit performed best. Unsurprisingly, the GTR+G+I model itself, which was predicted to have the best fit to the sequence data, was often outperformed by the simpler JC and K2P models. Note that the difference in performance between *Δ*_JC_ and the two other SR functions is greater for subsets that are more accurately reconstructed by the BIONJ-GTR approach (the lower bins). This appears to indicate that over-simplified distance methods are particularly beneficial when the sequence data conveys a stronger phylogenetic signal.

## Conclusions

In this paper we explored the basic properties of methods for estimating evolutionary distances, and studied how these properties affect the accuracy of distance-based phylogenetic reconstruction. We considered both the systematic bias and the stochastic noise (variance) of the distance estimators, and examined the tradeoff between these two factors. We focused on the common task of phylogenetic reconstruction under homogeneous substitution models. Assuming homogeneous models simplifies the analytical framework, since in such models each SR function is reduced to a univariate function of the evolutionary time *t* . However, obtaining accurate estimates of *t* is still a hard task in this setting, since the unit rate matrix is unknown. An SR function *Δ* is guaranteed to yield consistent reconstruction across *all* trees in a homogeneous model only if it is additive, meaning that it is a linear function of *t* . When *Δ* is not additive, it introduces a systematic bias in distance estimates, which we denoted here as *deviation from additivity* . Some SR functions are only additive in one homogeneous model, whereas others are additive across a wider collection of homogeneous models. This less constrained additivity is typically achieved at a price of increased estimation noise. We studied the tradeoff between “deviation from additivity” and “estimation noise” via a case study where the model tree is a homogeneous K2P-tree with an unknown ti-tv ratio *R* . In this case, Kimura’s distance formula *Δ*_K2P_ is always additive, while the less noisy Jukes Cantor’s formula, *Δ*_JC_, is additive only when $R=\frac{1}{2}$.

A study of this type requires a way to measure the deviation from additivity of a non-additive SR function *Δ* in a given range of distances [*t*_0_,*t*_1_]. To this end, we introduced the concept of affine-additive distance functions, and defined the deviation from additivity of *Δ* in [*t*_0_,*t*_1_] as the distance of *Δ* from its closest affine-additive function in [*t*_0_,*t*_1_]. We established a tight connection between this measure and statistical consistency of reconstruction (Lemma 2.7) and derived an upper bound for deviation from additivity in homogeneous models (Lemma 2.8). We applied these results in analyzing the deviation from additivity of *Δ*_JC_, and its effect on the accuracy of reconstructing homogeneous K2P-trees. We then showed, both analytically (in Section Deviation from additivity in homogeneous substitution models) and through experiments on simulated data sets (in Sections Performance of Non affine-additive SR functions in quartet resolution and Simulations on Hasegawa’s Tree), that, compared to *Δ*_K2P_, it is often better to use the non-additive but less noisy estimates of *Δ*_JC_, even when *R* is quite high. Somewhat surprisingly, we found this to be the case even when the tree being reconstructed has an “unfavorable” topology. Our experiments on bacterial gene sequences (Section Inferring trees from genomic sequences) also indicate that the simple and less noisy SR functions perform better on average than ones that are expected to better fit the true substitution process.

The framework presented in this paper implies a practical way for selecting SR functions which are likely to increase the accuracy of distance estimation. The practicality of the method is drawn from the fact that the criteria by which we select an SR function depend only a relatively crude information about the tree being reconstructed. For instance, in the case of a homogeneous K2P-tree, one can easily obtain from the input sequences rough estimates of both the ti-tv ratio *R* and the range of inter-leaf times [*t*_0_,*t*_1_]. These estimates can then be used to compare the expected accuracies of *Δ*_JC_ and *Δ*_K2P_ on the given input, and determine which of them is more likely to yield an accurate phylogeny. For quartets, a tight comparison can be made using the FC-based approach suggested in Section Fisher’s Criterion for Separability, and for larger trees, a cruder comparison can be made using a plot like the one presented in Figure 1b. A promising avenue of further research is to extend the FC-based approach to allow tighter prediction of reconstruction accuracy of trees spanning more than four taxa.

## Endnotes

^a^This is a WABI 2011 special issue invited paper. Extended abstract of this paper appeared in [47]. ^b^Typically, the unit rate matrix is assumed to be the one corresponding to one substitution per site. ^c^Many common distance-based algorithms, such as the Neighbor Joining (NJ) algorithm [31,32], are known to be robust in this sense. ^d^In a tree, edges which touch leaves are *external*, and all other edges are *internal*. ^e^Types A and B quartets represent the *Farris zone* and *Felsenstein zone*, resp. (see, e.g., [1], Chapter 9). ^f^We use here the square root of the criterion commonly used in the literature, because we prefer to think in terms of distances rather than squares of distances. This has no practical influence, since we use FC only for comparing between different choices, not for assessing the quality of a give choice.^g^ This ML estimate is obtained by a simple numerical method for maximizing the likelihood function (see, e.g., [1]).

## Appendix

## Tightness of Lemma 2.8

Let *f* (*t* ) be a (continuous) function on some interval [*t*_0_,*t*_1_]. We prove below that if *f * does not intersect its linear interpolation *At* + *B* in that interval, then $\mathrm{dev}(f,[t_{0},t_{1}])=\frac{1}{A}{\text{max}}_{t\in [t_{0},t_{1}]}\left\{\left|f(t)-\mathrm{At}-b^{*}\right|\right\}$. We use the following notations, conforming to the notations in the proof of Lemma 2.8:

$$\begin{array}{c} \psi (a,b,t)\;=\;f(t)-\mathrm{at}-b\\ \psi (a,b)=\underset{t\in [t_{0},t_{1}]}{\max}\left\{\left|\psi (a,b,t)\right|\right\}\;\psi (a)=\underset{b\in R}{\min}\left\{\psi (a,b)\right\}.\;\;\; \end{array}$$

### Lemma 2.9

Let *f* (*t* ) be a monotone increasing function in the interval [*t*_0_,*t*_1_] and let *At* + *B* be its linear interpolation in [*t*_0_,*t*_1_]. If either *f* (*t* )≥*At* + *B* for all *t* ∈[*t*_0_,*t*_1_] or *f* (*t* )≤*At* + *B* for all *t* ∈[*t*_0_,*t*_1_], then for all *a* >0, we have $\frac{1}{a}\psi (a)\;\geq \;\frac{1}{A}\psi (A)$.

### Proof

We prove the minimality of $\frac{1}{A}\psi (A)$ in the case where *f* (*t* )≥*At* + *B* for all *t* ∈[*t*_0_,*t*_1_]. The other case (where *f* (*t* )≤*At* + *B* for all *t* ∈[*t*_0_,*t*_1_]) can be proven in an identical fashion.

**Figure 7:**
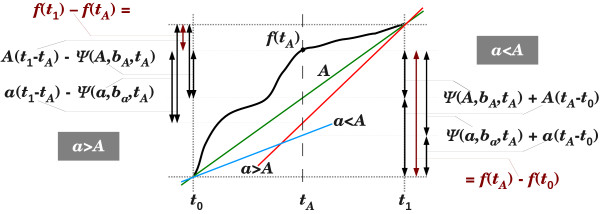
**Proof of Lemma 2.9.** A function **f(t)** is portrayed (bold) with its linear interpolation **At** + ***B*** =***At*** + ***B***_***A***_(green) in the interval [***t*_0_,*t*_1_**], s.t.***f*** (***t***)≥***At*** + ***B*** for all*t* ∈[***t*_0_**,***t*_1_**]. Equation (18) is illustrated for *a* <*A* on the right, and equation (19) is illustrated for *a* >*A* on the left.

For *a* >0, let *B*_*a*_be the maximum value of *B*^*′*^s.t. $\psi (a,b^{\prime },t)\geq 0$ for all *t* ∈[*t*_0_,*t*_1_]. Evidently, $\psi (a)=\frac{1}{2}\psi (a,b_{a})$. If the linear interpolation of *f* (*t* ) in [*t*_0_,*t*_1_] is given by *At* + *B*, then *B*_*A*_=*B* . We need to show that for every *a* >0, it holds that *Aψ* (*a*,*B*_*a*_)>*aψ* (*A*,*B*_*A*_). Let *t*_*A*_be a point in [*t*_0_,*t*_1_] s.t. *ψ* (*A*,*B*_*A*_,*t*_*A*_)=*ψ* (*A*,*B*_*A*_). Note that if *a* <*A*, then the two linear functions *At* + *B*_*A*_and *at* + *B*_*a*_intersect at (*t*_0_,*f* (*t*_0_)), and if *a* >*A*, then they intersect at (*t*_1_,*f* (*t*_1_)) (see Figure 7).

For *a* <*A*, we get the following equality (Figure 7; right):

(18) $$\begin{array}{c} \psi (A,b_{A},t_{A})+A(t_{A}-t_{0})\;=\;f(t_{A})\\ \;-f(t_{0})\;=\;\psi (a,b_{a},t_{A})+a(t_{A}-t_{0})\;. \end{array}$$

Hence, since *ψ* (*a*,*B*_*a*_)≥*ψ* (*a*,*B*_*a*_,*t* ) for every *t* ∈[*t*_0_,*t*_1_], and since *a* <*A*, we get

$$\begin{array}{c} \;\mathrm{a\psi}(A,b_{A},t_{A})+\mathrm{aA}(t_{A}-t_{0})\;<\;\mathrm{A\psi}(a,b_{a},t_{A})\\ \;+\mathrm{Aa}(t_{A}-t_{0})\Rightarrow \;\mathrm{a\psi}(A,b_{A})<\mathrm{A\psi}(a,b_{a}). \end{array}$$

Similarly, if *a* >*A*, we get the following equality (Figure 7; left)

(19) $$\begin{array}{c} A(t_{1}-t_{A})-\psi (A,b_{A},t_{A})\;=\;f(t_{1})\\ \;-f(t_{A})\;=\;a(t_{1}-t_{A})-\psi (a,b_{a},t_{A})\;, \end{array}$$

and *a* >*A* implies that

$$\begin{array}{c} \;\mathrm{aA}(t_{1}-t_{A})-\mathrm{a\psi}(A,b_{A})>\mathrm{Aa}(t_{1}-t_{A})\\ \;-\mathrm{a\psi}(a,b_{a})\;\Rightarrow \;\mathrm{a\psi}(A,b_{A})<\mathrm{A\psi}(a,b_{a})\;. \end{array}$$

□

## Competing interests

The authors declare that they have no competing interests.

## Authors’ contributions

All authors participated in discussing, formulating, and modulating the research. DD performed the simulations and experiments of Sections Simulations on Hasegawa’s Tree and Section Inferring trees from genomic sequences. IG and SM initiated and directed the research and drafted the manuscript. IY performed the analysis in Sections Deviation from additivity in homogeneous substitution models and Section Performance of Non affine-additive SR functions in quartet resolution and contributed to the ideas of the project. All authors contributed to the writing and editing of the manuscript, and all authors read and approved the final manuscript.
